# Correlation between SDF-1α, CD34 positive hematopoietic stem cells and CXCR4 expression with liver fibrosis in CCl4 rat model

**DOI:** 10.1186/s12876-023-02932-y

**Published:** 2023-09-21

**Authors:** Sara Abubakr, Noha M. Hazem, R.N Sherif, Adel Abdelmohdy Elhawary, Kamal G Botros

**Affiliations:** 1 Human Anatomy & Embryology Department, Faculty of Medicine, Mansoura University, Mansoura, Egypt; 2 Medical Biochemistry and Molecular Biology Department, Medical Experimental Research Center (MERC), Faculty of Medicine, Mansoura University, Algomhoria Street, Mansoura, 35516 Egypt; 3 Pathological Sciences Department, Fakeeh College for Medical Sciences, Jeddah, Saudi Arabia

**Keywords:** SDF- 1α, CD34, CXCR4, Hematopoietic stem cells, Liver fibrosis, Spontaneous reversion

## Abstract

**Background:**

One of the most frequent disorders is liver fibrosis. An improved understanding of the different events during the process of liver fibrosis & its reversibility could be helpful in its staging and in finding potential therapeutic agents.

**Aim:**

The goal of this research was to evaluate the relationship among CD34 + HPSCs, SDF-1α, and CXCR4 receptor expression with the percentage of the area of hepatic fibrosis.

**Materials and methods:**

Thirty-six male Sprague-Dawley rats were separated into the control group, liver injury group & spontaneous reversion group. The liver injury was induced by using 2 ml/kg CCl4 twice a week. Flow cytometric examination of CD34 + cells in the blood & liver was performed. Bone marrow & liver samples were taken for evaluation of the SDF-1α mRNA by PCR. Liver specimens were stained for histopathological and CXCR4 immuno-expression evaluation.

**Results:**

In the liver injury group, the hepatic enzymes, fibrosis area percentage, CXCR4 receptor expression in the liver, CD34 + cells in the blood and bone marrow & the level SDF-1α in the liver and its concentration gradient were statistically significantly elevated with the progression of the liver fibrosis. On the contrary, SDF-1α in the bone marrow was statistically significantly reduced with the development of liver fibrosis. During the spontaneous reversion group, all the studied parameters apart from SDF-1α in the bone marrow were statistically substantially decreased compared with the liver injury group. We found a statistically substantial positive correlation between fibrosis area and all of the following: liver enzymes, CXCR4 receptor expression in the liver, CD34 + cells in the blood and liver, and SDF- 1α in the liver and its concentration gradient. In conclusion, in CCl4 rat model, the fibrosis area is significantly correlated with many parameters in the blood, bone marrow, and liver, which can be used during the process of follow-up during the therapeutic interventions.

## Introduction

Hepatic fibrosis is primarily marked by an excessive buildup of extracellular matrix (ECM), particularly collagen fibers, and is a repair reaction to chronic liver damage brought on by numerous pathogenic causes [1]. The most frequent causes of liver fibrosis are alcohol misuse, chronic viral hepatitis, overweight, autoimmune hepatitis, metabolic disorders & cholestasis [2]. The ultimate progression of liver fibrosis to cirrhosis or even hepatocellular malignancy depends on the know-how to eliminate the causative pathogen [3].

The interactions among the CXCR4 chemokine receptor (CXCR4) and its ligand, stromal cell-derived factor 1α (SDF-1), which are the regulators of hematopoietic stem cells (HPSCs), are now in focus in current literature. SDF-1α is produced by osteoblasts, which are a specific kind of reticular cells that may be found in the endosteal niche as well as the vascular niche [4]. Hematopoietic stem and progenitor cells that express CXCR4 are attracted to and maintained in the bone marrow by the chemoattractant SDF-1α [5].

The main element regulating stem cell homing & migration out of the bone marrow is the chemokine SDF-1α. It is crucial for the BM’s stem cell seeding throughout its advancement via its receptor CXCR4 [6, 7]. Stem cells may be drawn to the site of damage by an elevation in SDF-1 concentrations, where they facilitate tissue regeneration and repair [8]. Several investigations have shown that SDF-1 encourages stem cell migration & homing to the damaged tissues [9, 10]. The mobilization of HPSCs and progenitor cells along the level gradient of SDF-1 is induced by elevated SDF-1 levels in the circulation [11].

Previous reports revealed that HPSCs have hepatic potential [12] and showed that hepatocytes are produced from the bone marrow of the recipient of a gender-mismatched bone marrow transplant at a high frequency that ranges from 4 to 7%. Many experimental and clinical studies have been carried out to gain improvement in comprehension of the effects of bone marrow stem treatment on patients who suffer from liver disease. For several years, the surface marker CD34 antigen was only used to determine the hematopoietic cells [13, 14]. The injured liver releases chemokines such as SDF-1α to participate in attracting bone marrow stem cells and their homing to the liver [15, 16].

Current research in experimental hepatic fibrosis models and clinical investigations has supported the hypothesis that liver fibrosis is reversible if the injury-causing stimulus is removed [17, 18]. A cascade of events occurs to initiate the process of reversion upon the withdrawal of the causative agent. The loss of fibrous scars and myofibroblasts through senescence and apoptosis, the decrease in cytokine levels, and the increase in collagenase activity are the initial events during the reversion of liver fibrosis [19, 20].

With the growing knowledge of liver fibrosis, novel substances with antifibrotic potential have surfaced and are being tested in clinical settings [21–23]. Identifying the illness’s state is crucial for selecting a course of treatment and determining the prognosis. The demand for more reliable & non-invasive techniques for the diagnosis and staging of hepatic fibrosis is developing even though liver biopsy is still the standard reference for determining the extent of liver fibrosis [24–26].

An improved understanding of the different events that occur throughout the process of liver fibrosis and its reversibility; SDF-1α in the bone marrow and liver, and CD34 + cells in the liver and the blood can illuminate our understanding of this process.

### Aim of the work

The current research was undertaken to evaluate the correlation between the CD34 + HPSCs in the liver & peripheral blood, SDF-1α in the liver and bone marrow and its concentration gradient, and CXCR4 receptor expression with the process of liver fibrosis progression and reversion in the CCl4 rat model using biochemical, histological, immunohistochemical, and molecular biological techniques.

## Materials and methods

### Sample size calculation

G*Power software (version 3.1.7.9) was utilized to compute the sample size. Based on a review of the literature [11, 27, 28] we predicted our parameters would have a big impact (as compared between the 5 study groups with ‘f’ = 0.7 or more). In one-way ANOVA research, samples of six rats from each of the six groups whose averages are to be compared are acquired. An F test with a significance threshold of 0.0500 and a total sample size of 36 participants provides 86% power to identify mean variations compared to the alternative of equal means. The effect size f = σm / σ, 0.7000, is used to describe how much the means varied.

### Animals used

The experiment was conducted on 36 adult male Sprague-Dawley rats with a median weight of “200–250” grams, purchased from the Mansoura Experimental Research Center (MERC) in Mansoura, Egypt. Rats were kept in metal cages with bedding made of softwood chips under constant temperature (23ºC ± 3) and relative humidity conditions. For 2 weeks before the experiment, the animals were given unrestricted access to conventional commercial feed, tap water, and a 12-hour light-dark cycle to acclimate and assure normal development and behavior. All rats were kept in the animal home in a specialized environment that was pathogen-free. This experiment was completed in the Faculty of Medicine being studied at the MERC on the campus of Mansoura University. All of the studies were performed in compliance with the rules provided by the National Institutes of Health (NIH) and the Institutional Review Board (IRB) for the care and use of laboratory animals. Additionally, the research was reported consistent with the criteria provided by ARRIVE (https://arriveguidelines.org). The Mansoura Faculty of Medicine’s Institutional Review Board gave its approval to the project (Approval No. MD/17.03.16).

### Chemicals used

Carbon Tetra Chloride (CCl4) for induction of liver fibrosis model (Sigma-Aldrich Cat. No. 289,116, Germany).

### Design of the experiment

Following a two-week acclimation period, the rats were split randomly into three groups utilizing Microsoft Excel’s standard = RAND () function: Control group (*n* = 12): rats got intraperitoneal (IP) injections of 2 mg/kg olive oil (solvent for CCL4) twice a week for 4 weeks (*n* = 6) & 8 weeks (*n* = 6). Chronic liver injury (CCL4-treated) group (*n* = 12): rats got IP injections of 2 mL/kg CCl4 soluble in olive oil (1:1) twice a week as previously indicated by Zhao et al. [29]. Six rats were chosen randomly and sacrificed at 4 weeks (4wks CCl4, *n* = 6) & 8 weeks (8wks CCl4, *n* = 6) from the first injection. Spontaneous reversion group (*n* = 12): rats got IP injections of 2 mL/kg CCl4 soluble in olive oil (1:1) twice a week for 8 weeks. Six rats were chosen randomly and sacrificed after 2 weeks (2wks reversion; *n* = 6) and 4 weeks (4wks reversion; *n* = 6) from the last injection of CCL4.

### Sample collection

Blood samples from the tail vein were taken at the appointed time for each group, after which the rats were given an IP administration of chloral hydrate (300 mg/kg) to put them to sleep before being dissected. Blood Samples: Blood samples were collected in Sangeetha evacuated tubes by direct left ventricle puncture for assessment of liver function tests (AST and ALT) and serum levels of SDF-1α. Bone Marrow Samples: Rat tibia and femur bone marrow served as the source of BMSCs for the flow cytometric examination of CD34 + cells. Separately, the femur and tibia of each leg were put in a 50-ml centrifuge tube containing antibiotics, DPBS (Dulbecco’s Phosphate Buffered Saline), and a petri dish with DME (Dulbecco’s Modified Eagles Media). Both bones’ metaphyseal areas were sliced, and then a needle was inserted into the medullary cavity to remove the bone marrow using DMEM and placing it in a 15-ml centrifuge tube. To concentrate the cells, the bone marrow was centrifuged for 5 min at 1000 rpm [30]. Liver Specimens: After opening the abdomen, the liver was carefully removed, preserved in 10% buffered formalin, and prepared for paraffin sectioning. Other fresh liver specimens were processed for PCR evaluation of the level of SDF-1 and flow cytometric analysis for CD34 + cells.

## Methods and staining techniques

### Biochemical tests

Assessment of liver enzymes The sera were produced by centrifuging blood for 10 min at 5000 g at 4 °C, coding them, and then utilizing clinical test kits from Elitech (UK) to assess the concentrations of alanine transaminase (ALT) and aspartate transaminase (AST) spectrophotometrically [31].

Histological examination of the liver: For histological analysis, transverse slices were cut at a thickness of 5–6 μm and stained with hematoxylin and eosin [32], Sirius red to assess the architectural alteration and collagen accumulation [33] and immunohistochemically with an anti-CXCR4 antibody stain. All sections were coded and examined in a double-blind manner by two different investigators. Immunohistochemistry for CXCR4 receptors [34]: To suppress endogenous peroxidase, tissue sections were first blocked in 10% normal goat serum for 30 min before being treated with Rabbit monoclonal anti-CXCR4 (1:500, ab124824; Abcam Corp., UK) at 4 degrees Celsius for an entire night. After that, the sections were incubated with 3% hydrogen peroxide at room temperature for thirty minutes. After being washed in PBS, the slides were then subjected to a treatment with a secondary antibody (anti-rabbit detection system; Boster, China) at 37 degrees Celsius for thirty minutes. This was followed by visualization with 3-diaminobenzidine and counterstaining with hematoxylin. Positive cells were those with brown or brownish-yellow particles that were clearly visible in the cytoplasmic nucleus. Instead of the main antibody, portions under control were treated with PBS. The sections were coded and examined blindly by two different investigators.

### Evaluation of CXCR4 expression and Sirius red area percentage

Quantitative assessment of the percentage of liver fibrosis and optical density of CXCR4 positive expression was performed with morphometry on sections processed with 0.1% Picro Sirius red and CXCR4 immuno-stained sections, respectively. According to Traber et al. [35], nine randomly selected photos were captured on each of the four stained slides per animal utilizing the Olympus® SC100 digital camera mounted on the Olympus® CX41light microscope. The National Institutes of Health, Bethesda, Maryland, USA, provided the software, which was used for morphometric investigation. To assess the existence and degree of CXCR4 expression in the DAB pictures and for data collection, ImageJ v2.35 (NIH) was utilized. By applying a histogram profile to the deconvoluted DAB picture using the H-DAB-vector, an ImageJ plugin was used to assess the cytoplasmic staining and produced three distinct images in the colors green, brown, and blue. The DAB pictures were calibrated by calculating the average intensity of five distinct, non-overlapping sections of the stained tissue [36]. The intensity numbers were converted into OD using the formula below: OD = log (Max intensity/average intensity), where the max intensity is 250 and the mean intensity is the mean gray value.

Gene Expression by Quantitative Real-Time Polymerase Chain Reaction (qRT-PCR) for Evaluation of SDF-1 Levels in Both Blood and Liver: SDF-1 gene expression in tissue homogenates of liver and bone marrow samples was quantified by qRT-PCR. The procedure involves total RNA isolation, evaluation of the extracted RNA’s quality, reverse transcription, and relative quantitation of gene expression [37]. Tissue samples were homogenized in buffer RLT using liquid nitrogen with a mortar and pestle. Whole-RNA isolation was done according to the RNeasy mini column (Qiagen, Germany) and the manufacturer’s manual. Thermo Scientific’s NanoDrop 2000 was used to measure the amount of RNA (USA). Using the Thermo Scientific Maxima First Strand cDNA synthesis kit for qRT-PCR (catalog no. 205,311; Thermo Scientific, Rockford, USA) and the procedure outlined in [38], reverse transcription of 1ug of RNA was carried out. Then, cDNA templates were used for running the PCR reaction on a real-time PCR instrument (USA: Integrated Biosystem 7500). B-actin was used as a housekeeping gene. Primer sets were synthesized by Invitrogen (Thermo Scientific, Rockford, USA), and their sequences are listed in Table 1. To determine relative gene expression levels, [39] described an approach.

**Table 1:** The primer pairs utilized gene sequences

**SDF1 (NM_022177.3)**	Forward primer	5-CTCTGCATCAGTGACGGTAAGC-3
	Reverse primer	5-GGATTTTCAGATGTTTGACGTTGG-3
**B-actin (NC_005111)**	Forward primer	5′-GAACCCTAAGGCCAACC-3′
	Reverse primer	5′-TGTCACGCACGATTTCC-3′.

### Flow cytometric analysis of CD + 34 cells in bone marrow & liver tissue

A FACS-caliber flow cytometer (Becton Dickinson, Sunnyvale, CA, USA) was used at the Mansoura Children’s Hospital. According to Grogan et al. [40], fresh tissue samples were transported in isotonic saline. 0.1 M tris (hydroxymethyl aminomethane), 0.07 M sodium chloride (ADWIC), and 0.005 M EDTA at PH 7.5 were used to wash the tissue. The cells were centrifuged, fixed in ice-cold 96–100% ethanol (BDH), and then incubated for at least 30 min in the dark at room temperature with 1 g/ml Anti-CD34 PE. For examination by the flow cytometer, the cells were washed and then resuspended in ice-cold PBS, 10% FCS, and 1% sodium azide.

### Statistical analysis

With the aid of the 2019 release of IBM Corp.‘s SPSS program, data were input and examined. Armonk, NY: IBM Corp., IBM SPSS Statistics for Windows, Version 26.0. Shapiro-test Wilk’s was utilized to determine the data’s normality, and boxplots were examined to look for any noteworthy outliers. As the data was regularly distributed across all variables and groups and lacked any appreciable outliers, it was reported as the mean and standard error (SE). To compare normally distributed quantitative data across the five groups, a one-way ANOVA was used. Univariate GLM (Partial eta squared [η2] and G*Power software (Cohen’s f) were used to determine the effect size. The Tukey HSD tests were used to compare two things in pairs. Results for any test employed were deemed statistically significant if the *p*-value ≤ 0.050. The direction & strength of the linear link between two quantitative variables were examined using Pearson’s and Spearman’s correlations. There was a reported correlation coefficient (r). Negative values signify a bad correlation, whereas positive ones indicate a good relationship. If the r value is < 0.1, there is no correlation; 0.1 to 0.3, a mild association; 0.3 to 0.5, a medium relationship; and > 0.5, a strong link.

## Results

No substantial variation was noted between the control group at the 4th and 8th weeks in any of the studied parameters, so their results were summarized as one group.

The liver transaminases AST, ALT, and AST/ALT ratio are revealed in Table 2. Administration of CCl4 for 4 and 8 weeks led to a substantial progressive elevation of the transaminase level contrasted with the control group. Meanwhile, cessation of CCl4 injection was followed by a progressive and significant reduction in transaminase levels in the reversion group contrasted with the 8 W CCl4-treated group, although still significantly high contrasted with the control group.

The AST/ALT ratio showed a statistically substantial elevation in the 4wks and 8wks CCL4-treated groups compared to the control group. The 4wks reversion group stated a statistical decrease in the AST/ALT ratio compared with the 8wks CCL4 group, and the ratio was not substantially different from that of the control group. Meanwhile, the AST/ALT ratio of the 2wks reversion group did not show a substantial difference compared with the 8wks CCl4-treated group (Table 2).

**Table 2:** The serum level of the liver Enzymes in the 5 study groups

**Liver transaminases** **(median±SE)**	**Group**			**One-Way ANOVA**		**Effect Size**	
	**Control**	**4wks CCL4**	**8wks CCL4 **	**F**	***p*** **-value**	**Partial η2 (a)**	**Cohen’s f**
**AST (IU/L)**	7.82 ± 0.60	124.33 ± 2.56^ab^	306.83 ± 6.36^a^	1436.809	**< 0.001**	0.995	14.10674
**ALT (IU/L)**	7.67 ± 0.45	51.13 ± 2.12^ab^	124.5 ± 3.65^a^	579.658	**<0.001**	0.987	8.713385
**AST/ALT Ratio**	1.02 ± 0.07	2.46 ± 0.13^a^	2.47 ± 0.07^a^	74.790	**<0.001**	0.909	3.16054
		**2wks reversion**	**4wks reversion**	**F**	***p*** **-value**	**Partial η2 (b)**	**Cohen’s f**
**AST (IU/L)**		188.00± 3.37^ab^	38.233± 0.55^abc^	1470.086	**<0.001**	0.995	14.10674
**ALT (IU/L)**		84.38±3.99^ab^	27.15±0.94^abc^	376.226	**<0.001**	0.983	7.604178
**AST/ALT Ratio**		2.25± 0.10^a^	1.42± 0.06^b^	77.893	**<0.001**	0.921	3.414415

Partial η2 (a) is a measure of effect size between the groups (control and 4& 8wks CCl4) created by Univariate analysis in a general linear model (GLM)

Partial η2 (b) is a measure of effect size between the groups (control, 8wks CCl4, and 2 & 4wks reversion) created by Univariate analysis in a general linear model (GLM)

^a^Substantial variation VS control group

^b^substantial variation VS 8wks CCl4 group

^c^substantial variation VS 2wks reversion group

**Fig. 1:**
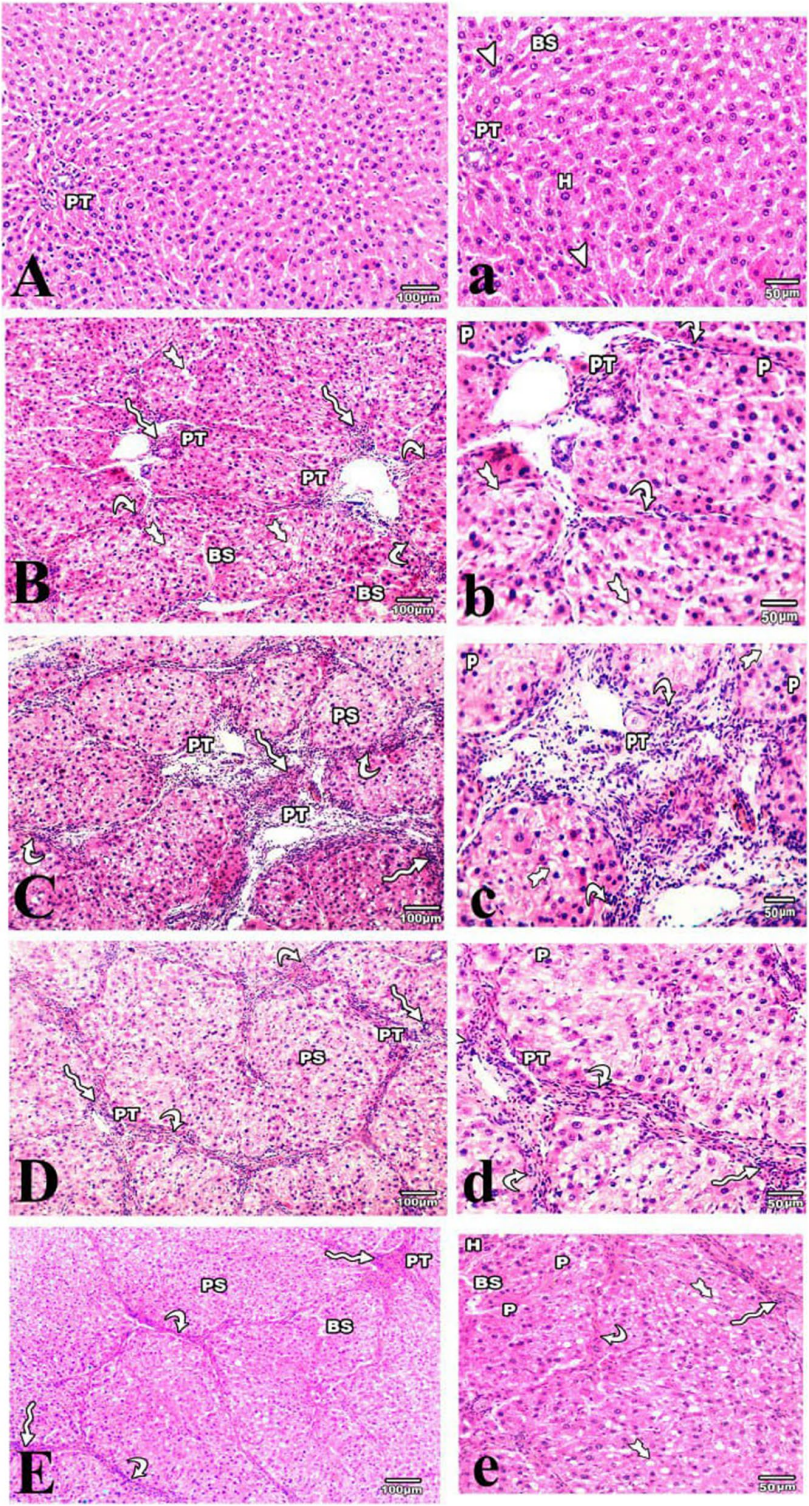
H & E-stained liver sections of the studied groups. **A**, **a** (control group): normal hepatic architecture with classic hepatic lobules formed of anastomosing cords of acidophilic hepatocytes (H), separated with blood sinusoids (BS) and some hepatocytes are binucleated (arrowheads). The portal tract (PT) is seen at the periphery of the lobule. **B**, **b** & **C**, **c** (4 & 8 wks CCl4 treated groups respectively): showing micro and macrovesicular steatosis (tailed arrows), pyknotic nuclei (P), portal tract (PT) infiltrated with mononuclear inflammatory cells (winding arrows), fibrous septa (curved arrows) radiating from the portal tract. Dilated blood sinusoids (BS) in **B** and pseudo lobules (PS) in **C** could be also seen. **D**, **d** & **E**, **e** (spontaneous reversion group 2 & 4 wks respectively): showing portal tract (PT), infiltrated with mononuclear inflammatory cells (winding arrows) with fibrous septa (curved arrow), pseudolobules (PS) and some pyknotic nuclei (P). In **e** some hepatocytes (H) appear normal while others still show degenerative changes; micro and macrovesicular steatosis (tailed arrows) & dilated blood sinusoids (BS) could be also seen

The histological appearance of the control liver revealed typical architecture with no apparent histological abnormalities. The liver sections were formed of the classic hepatic lobules, with cords of hepatocytes having acidophilic cytoplasm and central rounded vesicular nuclei radiating from the central vein. The portal triad was seen at the periphery of the lobules. Hepatic sinusoids with endothelial and Kupffer cell linings divided the hepatocyte cords. The interlobular septa were indistinct (Fig. 1A, a).

Administration of CCl4 for 4 weeks caused degenerative changes in the liver cells’ micro- and macro-vesicular steatosis and some pyknotic nuclei. The portal areas were thickened and infiltrated with a large number of mononuclear inflammatory cells. The amount of fibrous tissue in the portal vein and its surroundings increased. The normal liver architecture was retained, although incomplete fibrous tissue septa could be observed extending from the portal tract to the edges of the hepatic lobules (Fig. 1B, b).

Prolonged administration of CCl4 for 8 weeks produced marked degenerative changes with micro and macrovesicular steatosis and many pyknotic nuclei, dilatation of the blood sinusoids, and marked thickened and inflammatory cellular infiltration of the portal tract with thick fibrous tissue surrounding the hepatic pseudo-lobules, causing loss of the normal liver architecture (Fig. 1 C, c).

The liver Sect. 2 weeks following CCl4 cessation showed degenerative changes, especially at the periphery of the hepatic lobules, in addition to thickening and inflammatory cellular infiltration of the portal tracts with thick fibrous tissue septa and pseudo-lobules of varying shape (Fig. 1D, d).

On the other hand, partial improvement in hepatic architecture in the form of a few degenerative changes of the hepatocytes with a few mononuclear inflammatory cells in the portal tracts and some dilated sinusoids was observed 4 weeks following CCl4 cessation. Few fibrous connective tissue fibers were seen in the portal tracts, with thin bridging septa surrounding the pseudo-lobules (Fig. 1E, e).

**Fig. 2:**
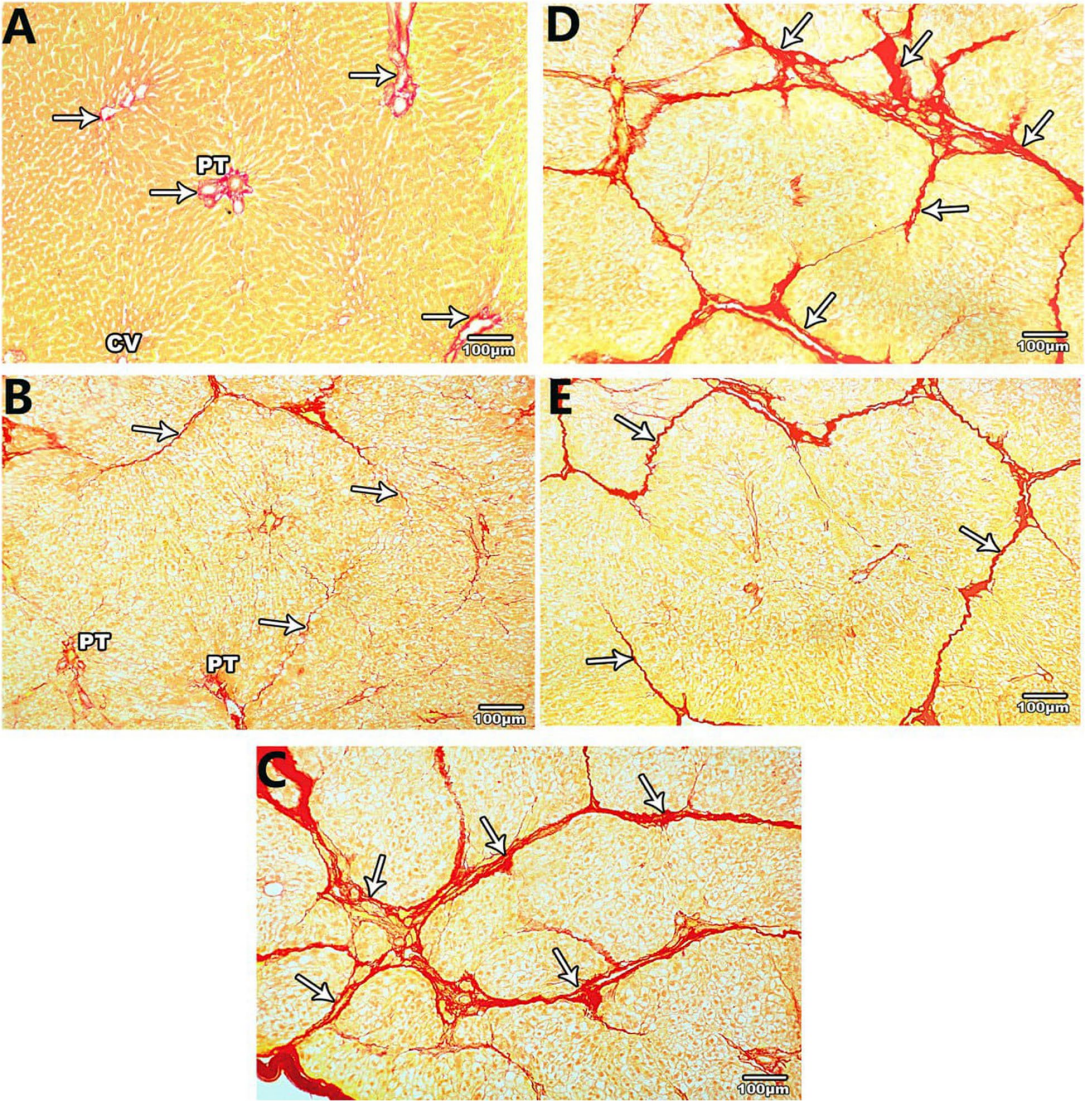
Sirius red-stained sections of the liver of the control, 4 and 8 wks CCl4 treated, 2 and 4 wks spontaneous reversion groups (A, B, C, D, and E respectively). **A**: scanty collagen fibers (arrows) around the central vein (CV) and in the portal tract (PT), **B**: thin fibrous septa (arrows) incompletely surrounding hepatic lobules, **C** & **D**: thick well developed fibrous septa (arrows) surrounding pseudo lobules, **E**: thin fibrous connective tissue septa (arrows) are seen surrounding the pseudo lobules

A few scanty, thin collagen fibres were observed in the portal tracts, around the central vein, and in the wall of the sinusoids of the liver of the control group (Fig. 2A). An increased amount of collagen fibers in the portal tract & around the central vein was observed in the CCL4 treatment group (Fig. 2B and C). Loss of hepatic architecture with pseudo-lobules surrounded completely by fibrous tissue septa with well-developed fibrous septa was observed in the 8-week CCL4 group (Fig. 2C). Liver sections two weeks after cessation of CCL4 injection did not show a variance from those of the 8-week CCL4 group. However, the septa became thinner and incompletely surrounded the hepatic pseudo-lobules 4 weeks after CCL4 cessation (Fig. 2D and E).

In the control group, the area percentage of the collagen fibers was 1.14%±0.19. The fibrous tissue area percentage was statistically substantially different in the five groups. The fibrous tissue area% showed a progressive statistically substantial increase in the 4 and 8 weeks CCl4 treated groups contrasted with the control group. In addition, the fibrous tissue area % was progressively statistically substantially reduced in the 2- and 4-weeks reversion groups contrasted with the 8 weeks CCl4 treated group, while still showing substantial enhancement contrasted with the control group (Table 3).

**Table 3:** The fibrosis area % and CXCR4 optical density in the 5 study groups

	**Group**			**One-Way ANOVA**	**Effect Size**		**One-Way ANOVA**
	**Control**	**4wks CCL4**	**8wks CCL4 **	**F**	***p*** **-value**	**Partial η2 (a)**	**Cohen’s f**
**Fibrosis area %** **(mean±SE)**	1.14 ± 0.19	9.20 ± 0.27^ab^	19.37 ± 0.69^a^	430.380	**< 0.001**	0.983	7.604178
**CXCR4 optical density** **(mean±SE)**	0.067 ± 0.004	0.129 ± 0.007^ab^	0.201 ± 0.009^a^	35.530	**<0.001**	0.826	2.17879
		**2wks reversion**	**4wks reversion**	**F**	***p*** **-value**	**Partial η2 (b)**	**Cohen’s f**
**Fibrosis area %** **(mean±SE)**		15.82± 1.29^ab^	11.76 ± 0.53^abc^	102.371	**<0.001**	0.939	3.923448
**CXCR4 optical density** **(mean±SE)**		0.095 ± 0.006^ab^	0.081 ± 0.013^abc^	49.674	**<0.001**	0.882	2.733967

Partial η2 (a) is a measure of effect size between the groups (control and 4& 8wks CCl4) created by Univariate analysis in a general linear model (GLM).

Partial η2 (b) is a measure of effect size between the groups (control, 8wks CCl4, and 2 & 4wks reversion) created by Univariate analysis in a general linear model (GLM).

^a^Substantial variation VS control group

^b^substantial variation VS 8wks CCl4 group

^c^substantial variation VS 2wks reversion group

**Fig. 3:**
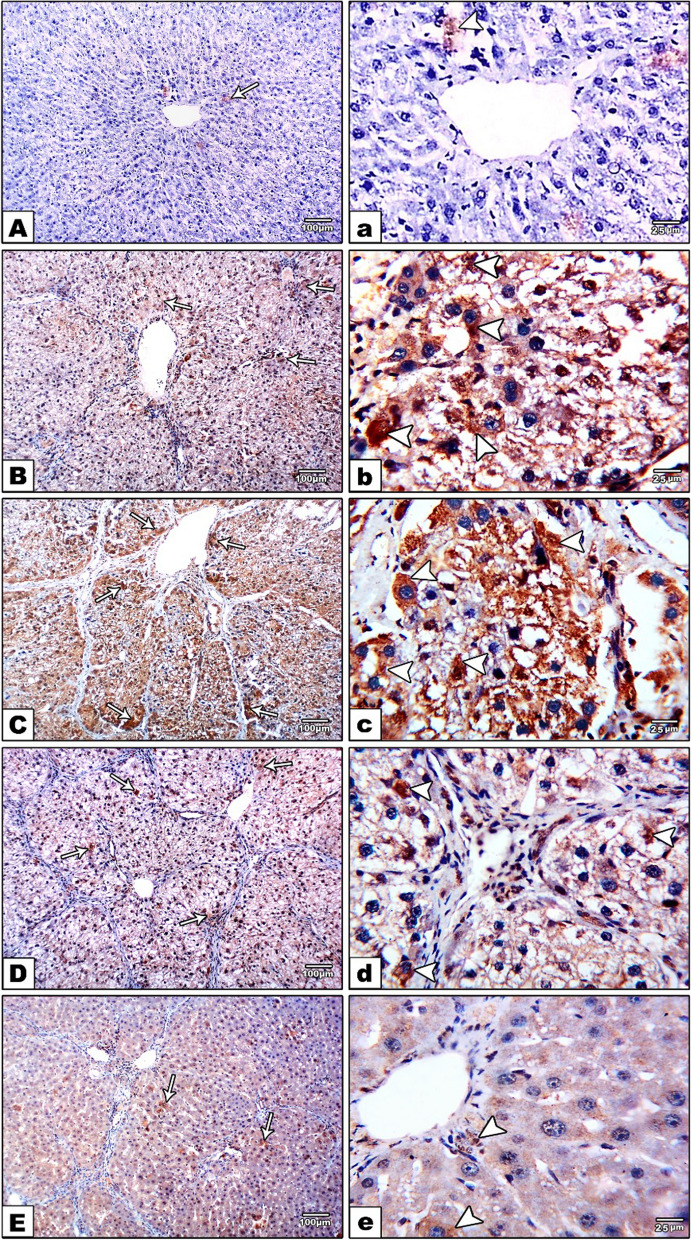
CXCR4 immunostained sections of the studied groups. **A**, **a** (control Group): minimal immune expression for CXCR4 (arrow), the reaction is cytoplasmic (arrowhead). **B**, **b** (4wks CCl4 treated group): positive immune reactivity in the portal tract and hepatic lobule (arrows), the reaction is mainly cytoplasmic (arrowheads). **C**, **c** (8wks CCl4 treated group): strong positive immune reactivity in the portal tract and scattered all over the hepatic lobule (arrows), the reaction is both cytoplasmic & nuclear (arrowheads). **D**, **d** (spontaneous reversion group 2wks): positive immune reactivity in the portal tract and hepatic lobule (arrows), the reaction is both cytoplasmic & nuclear (arrowheads). **E**, **e** (spontaneous reversion group 4wks): few positive cells are seen scattered in the portal tract and at the periphery of the hepatic lobules (arrows), the reaction is mainly cytoplasmic (arrowheads)

The liver sections of the control group showed minimal cytoplasmic expression within hepatocytes (Fig. 3A, a). Positive cytoplasmic CXCR4 reaction was observed mainly in the cells of the portal tract and at the periphery of the hepatic lobule in the 4 weeks CCL4-treated group (Fig. 3B, b). In addition, prolongation of the period of CCl4 administration for 8 weeks was associated with strong positive cytoplasmic and nuclear CXCR4 immunoreactivity in the portal tract and all over the hepatic lobule (Fig. 3 C, c). Two weeks after stopping the CCL4 injection, some positive cytoplasmic and nuclear CXCR4 reactivity was observed in a few cells all over the hepatic lobule (Fig. 3D, d). Meanwhile, only a few scattered positive cells at the periphery of the hepatic lobule were observed in the 4-week reversion group (Fig. 3E, e).

There was a statistically significant variance in CXCR4 positive expression amongst the 5 groups, as revealed by Pairwise comparison (Tukey HSD tests). The optical density of CXCR4 positive expression was statistically significantly higher in the CCl4 treatment group for 8 weeks contrasted with the CCl4 treated group for 4 weeks and the control group. Moreover, the optical density of the positive expression was statistically significantly lower in the reversion group contrasted with the 8-week CCl4-treated group, although still significantly high contrasted with the control group (Table 3).

**Fig. 4:**
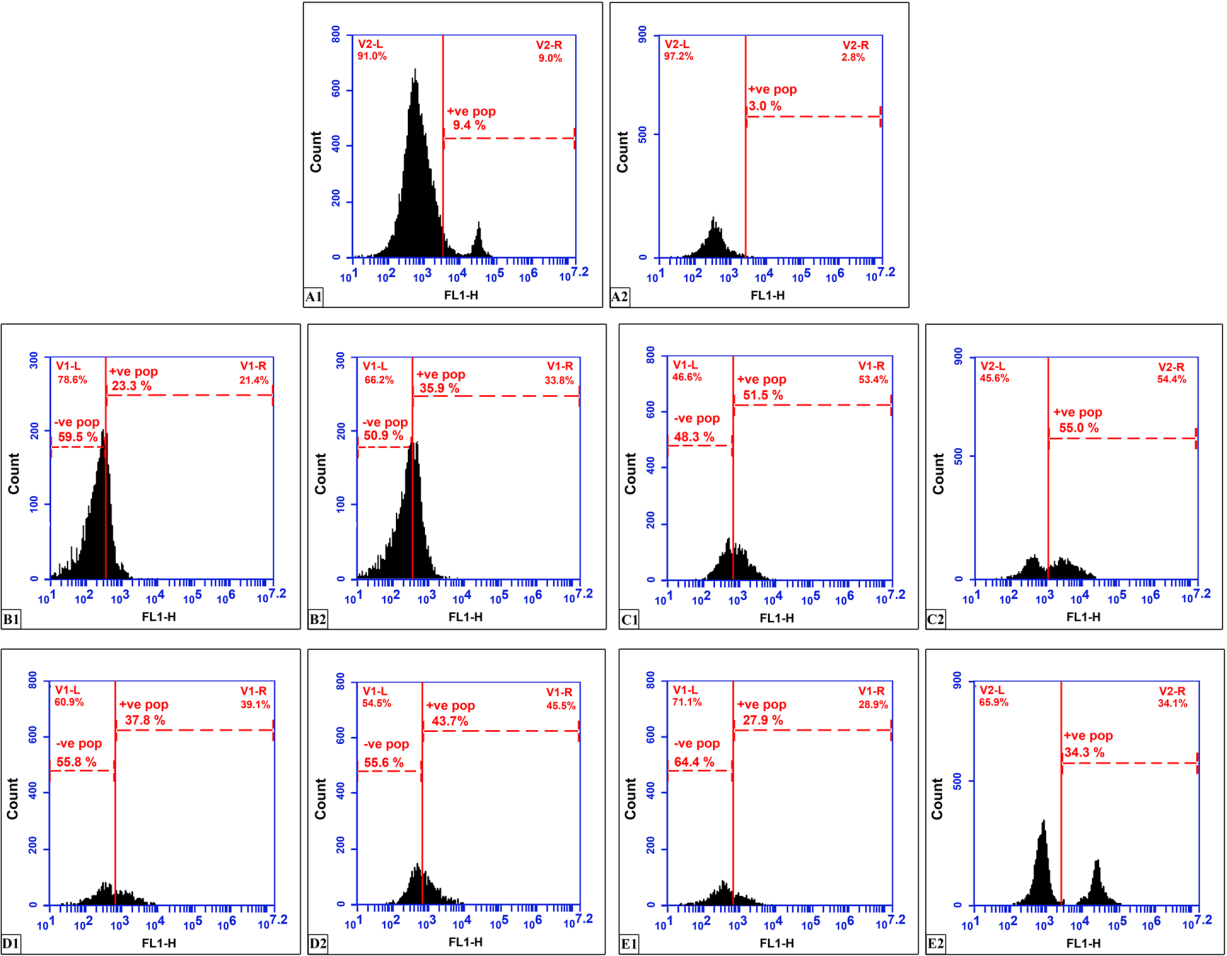
Flow cytometry for CD34 + in the blood (A1, B1, C1, D1 and E1) of the control, 4 and 8wks CCl4 treated, 2 and 4wks spontaneous reversion groups respectively and in the liver (A2, B2, C2, D2 and E2) of the control, 4 and 8wks CCl4 treated, 2 and 4wks spontaneous reversion groups respectively

In the control group, the percent of CD34 + cells in the peripheral blood and the liver was 8.98%±0.51 and 3.1%±0.09 respectively (Fig. 4A1, A2; Table 4). A significant progressive rise in the percentage of CD34 + ve cells was observed in both the peripheral blood and the liver in the 4 and 8-week CCl4-treated groups compared with the control group (Fig. 4B1, B2, C1, C2; Table 4).

Two and four weeks following CCl4 treatment stopping was accompanied by a significant progressive decrease of the percent of CD34 + cells in both the peripheral blood and the liver compared with rats sacrificed immediately following CCl4 treatment, although still significantly high compared with the control group (Fig. 4D1, D2, E1, E2; Table 4).

In the control group, the RQ of SDF-1α expression in the bone marrow and liver was 19.12 0.19 and 1.11 ± 0.004, respectively (Table 4). In the bone marrow, the RQ of SDF-1α showed a progressive statistically significant decrease in the 4 and 8 weeks CCl4-treated groups contrasted with the control group. Moreover, the RQ of SDF-1α was substantially elevated two weeks and four weeks after stopping CCl4 treatment compared with CCl4 for 8 weeks, although still substantially low compared with the control group. Furthermore, the liver samples revealed that CCL4 administration for 4 and 8 weeks caused a progressive and significant elevation of the RQ of SDF-1α contrasted with the control group. Moreover, in the two- and four-week reversion groups, the RQ of SDF-1α was progressively lower than that of the 8-week CCl4-treated group, while still substantially high contrasted with the control group (Table 4).

In the control group, a negative concentration gradient of SDF-1α expression toward the bone marrow was observed (Table 4). In the CCL4-treated group, a significant progressive increase in the concentration gradient toward the liver was determined in 4 and 8 weeks contrasted with the control value (Table 4).

In the spontaneous reversion group, the concentration gradient was positive towards the liver. A progressive and significant decrease of the concentration gradient in the 2- and 4-week spontaneous reversion groups was detected compared to the 8 W CCl4-treated group, although it was still significantly higher compared with the control group (Table 4).

**Table 4:** CD34+ % & SDF-1 α mRNA expression and concentration gradient in the 5 study groups

	**Group**			**One-Way ANOVA**		**Effect Size**	
	**Control**	**4wks CCL4**	**8 wks CCL4 **	**F**	***p*** **-value**	**Partial η2 (a)**	Cohen’s f
**CD34+ % in blood** **(mean±SE)**	8.98 ± 0.21	23.72 ± 0.23^ab^	50.65 ± 0.44^a^	4549.402	**< 0.001**	0.998	22.33831
**CD34+ % in liver** **(mean±SE)**	3.1 ± 0.37	35.08 ± 0.41^ab^	55.05 ± 0.23^a^	9331.564	**<0.001**	0.999	31.60696
**SDF-1 α mRNA gene expression in bone marrow** **(mean±SE)**	19.12 ± 0.19	8.62 ± 0.21^ab^	2.36 ± 0.15^a^	2044.276	**<0.001**	0.996	15.77973
**SDF-1 α mRNA gene expression in liver** **(mean±SE)**	1.11 ± 0.004	13.0 ± 0.06^ab^	19.33 ± 0.19^a^	6433.868	**<0.001**	0.999	31.60696
**SDF-1 α concentration gradient** **(mean±SE)**	-18 ± 0.19	4.38 ± 0.18^ab^	17 ± 0.17^a^	9480.891	**<0.001**	0.999	31.60696
		**2wks reversion**	**4wks reversion**	**F**	***p*** **-value**	**Partial η2 (b)**	**Cohen’s f**
**CD34+ % in blood** **(mean±SE)**		37.5 ± 0.50^ab^	26.37 ± 0.58^abc^	1508.696	**<0.001**	0.996	15.77973
**CD34+ %** ** in liver** **(mean±SE)**		42.8 ± 0.46^ab^	34.57 ± 0.51^abc^	3789.609	**<0.001**	0.998	22.33831
**SDF-1 α mRNA gene expression in bone marrow** **(mean±SE)**		5.69 ± 0.16^ab^	8.22 ± 0.26^abc^	1394.804	**<0.001**	0.995	14.10674
**SDF-1 α mRNA gene expression in liver** **(mean±SE)**		15.04 ± 0.02^ab^	11.79 ± 0.34^abc^	1560.472	**<0.001**	0.996	15.77973
**SDF-1 α concentration gradient** **(mean±SE)**		9 ± 0.15^ab^	4 ± 0.59^abc^	2048.795	**<0.001**	0.997	18.23001

Partial η2 (a) is a measure of effect size between the groups (control and 4wks & 8wks CCl4) created by Univariate analysis in a general linear model (GLM).

Partial η2 (b) is a measure of effect size between the groups (control, 8wks CCl4, and 2& 4wks reversion) created by Univariate analysis in a general linear model (GLM).

^a^Substantial variation VS control group

^b^substantial variation VS 8wks CCl4 group

^c^substantial variation VS 2wks reversion group

**Fig. 5:**
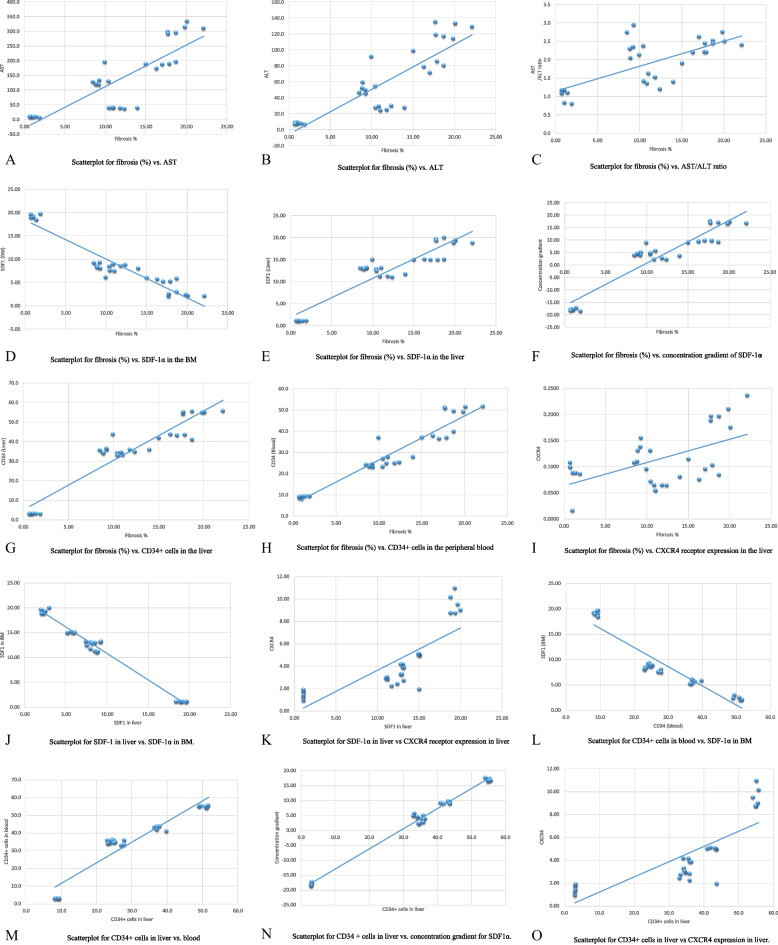
Scatterplots for correlations between variable parameters in the study

A statistically substantial positive correlation of large strength between the fibrosis area percentage in the liver versus liver transaminases level and ratio (Fig. 5A and B C and Table 5), CXCR4 receptor expression in the liver (Fig. 5I; Table 5), CD34 + cells (in both blood and liver) (Fig. 5G H and Table 5), SDF-1α mRNA level in the liver (Fig. 5E; Table 5), and its concentration gradient (Fig. 5F; Table 5) was detected. On the contrary, a statistically substantial negative correlation of large strength between the fibrosis area percentage and SDF-1α mRNA level in the bone marrow (Fig. 5D; Table 5) was detected. A statistically substantial positive correlation of large strength was observed between SDF-1α mRNA level in the liver and CXCR4 receptor expression in the liver (Fig. 5K; Table 5), and a statistically substantial negative correlation of large strength was observed between SDF-1α mRNA level in the liver and its level in the bone marrow (Fig. 5J; Table 5). Finally, a statistically substantial negative correlation of large strength between CD34 + cells in the blood and SDF-1α mRNA level in the bone marrow (Fig. 5L; Table 5) while a statistically substantial positive correlation of large strength between CD34 + cells in the liver and CD34 + cells in the blood (Fig. 5M; Table 5), concentration gradient for SDF-1α mRNA (Fig. 5N; Table 5), and CXCR4 receptor expression in the liver (Fig. 5O; Table 5).

**Table 5:** Correlation between studied parameters

**Parameters pair**		**Coefficient**	***p-value***
**Fibrosis (%)**^a^	**AST**	0.938	**< 0.001**
	**ALT**	0.909	**< 0.001**
	**AST/ALT ratio**	0.844	**< 0.001**
	**CXCR4 expression (liver)**	0.929	**< 0.001**
	**CD34 + cells (liver)**	0.885	**< 0.001**
	**CD34 + cells (Blood)**	0.838	**< 0.001**
	**SDF1 (liver)**	0.912	**< 0.001**
	**SDF1 (BM)**	-0.871	**< 0.001**
	**Concentration gradient**	0.901	**< 0.001**
**SDF1 in liver**	**SDF1 (BM)**	-0.992	**< 0.001**
	**CXCR4 expression (liver)**	0.805	**< 0.001**
**CD34 + in blood**	**SDF-1 (BM)**	-0.932	**< 0.001**
**CD34 + cells in the liver**	**CD34 + cells in the blood**	0.947	**< 0.001**
	**Concentration gradient for SDF1**	0.996	**< 0.001**
	**CXCR4 expression in liver**	0.806	**< 0.001**

The test of significance is Pearson’s or ^a^Spearman’s correlation test

Pearson’s and Spearman’s correlations were performed between the following studied parameters: liver function tests, SDF-1α in the liver and bone marrow, level gradients between SDF-1α in the liver and bone marrow, liver fibrosis percentage, CXCR4 receptor expression in the liver, and CD34 + cells in the blood and liver (Table 5)

## Discussion

Liver fibrosis incidence has been increasing gradually [41]. It is seen as a healing reaction that, although initially helpful in that it helps contain the harm by way of a reversible process, eventually advances to severe fibrosis or cirrhosis, which may be irreversible and cause reduced liver function and consequent death. [42–45].

The potential for established liver fibrosis to be spontaneously reversed has been proven in experimental rat models [20, 46–48]. Rats exposed to carbon tetrachloride after 4 to 8 weeks experience fibrosis, yet they can return to almost normal morphology if the exposure is stopped. On the other hand, cirrhosis develops after an injury that is left untreated for a longer period (12 weeks) and only partially reverses with the remodeling of a micro-nodular to a macro-nodular architecture [47]. A cascade of events occurs to initiate the process of reversion upon the withdrawal of the causative agent. The loss of fibrous scars and myofibroblasts through senescence and apoptosis, the decrease in cytokine levels, and the increase in collagenase activity are the initial events during the reversion of liver fibrosis [19, 20].

Current research in experimental hepatic fibrosis models and clinical investigations has supported the hypothesis that liver fibrosis is reversible if the injury-causing stimulus is removed [17, 18]. We have investigated the capability of spontaneous reversion of liver fibrosis in a rat model without adding olive oil, the vehicle for CCl4, to avoid any interference from any substance or drug as in previous studies by (Muriel 2005 and Pan 2007) who examined the process of spontaneous reversion of liver fibrosis in CCl4 rat models following discontinuation of CCl4 without giving the vehicle during such period [48, 49].

In the current study, we used CCl4 to induce liver fibrosis to examine the relation between multiple parameters that change with liver fibrosis, such as the serum liver transaminases enzymes, SDF-1α mRNA expression, percentage of CD34 cells, and CXCR4 expression, as a preliminary step to explore changes in which of them is closely correlated with the fibrosis area percentage. This might be useful to be used in the future in the development of a score for follow-up of liver fibrosis.

CCl4 induces hepatic injury when given at repetitively low doses, causing hepatocyte damage via free radical production, lipid peroxidation, increased oxidative stress, HSC and Kupffer cell activation, and TGF-B-1 upregulation [50, 51].

In the present study, CCl4 repetitive administration induced progressive and significant elevation of liver transaminases, progressive fatty changes, and necrosis of the hepatocytes, accompanied by inflammatory infiltration, leading finally to extensive fibrosis with distorted liver architecture after 8 weeks of injection, with a significant increase in the fibrous tissue area percentage in agreement with [52–54].

The transaminases are considered markers for liver cell inflammation [55, 56] and they leak out into the blood once the hepatic injury occurs [57] which is why they dramatically increased after CCl4 treatment.

The recovery of hepatic function and remodeling of the excess matrix are possible under certain circumstances [20, 58]. The discontinuation of CCL4 treatment has reversed the enzyme activities to normal values [49]. In agreement with [53], stopping the CCl4 injection was followed by a substantial reduction in the fibrosis area, along with improvement of the pathological changes in the liver and serum transaminases levels.

The kind of damage and how it affects hepatocytes, liver progenitor cells, and perhaps extrahepatic progenitor cells like those in the bone marrow define the cellular response to the liver injury. Our work illustrates the complex correlation between the different phenomena occurring during chronic liver injury induced by CCl4, such as the level of SDF-1α in BM and liver with its concentration gradient, CD34 in peripheral blood and liver, and CXCR4 receptor expression in the liver.

HPSCs are present in the BM niche in mammals [59], forming the majority of stem cells [60]. The hematopoietic cells possess a significant impact on experimental animal models of liver illness as well as on volunteers with chronic hepatic disorders [13, 14].

Through a variety of adhesion molecule interactions, HPSCs are hypothesized to be connected to osteoblasts, other stromal cells, and the ECM in this stem cell niche. The CXCR4 and SDF-1α, its ligand, interact with each other most significantly in the HPSC niche 4. HPSCs have CXCR4 receptors, and SDF-1α chemo attracts and keeps them in the bone marrow [61]. Under constant conditions, a small number of HPSCs continuously exit the BM via the CXCR4/SDF-1α axis, enter the tissues, and then return to the BM through the blood or lymphatic system [62]. SDF-1α and its receptor, CXCR4, are implicated in chemotaxis [63, 64], homing [65, 66], and survival of hematopoietic stem cells [67]. SDF-1α/CXCR4 may be involved in the retention of hematopoietic stem cells within the marrow [68]; this suggests that changing the SDF-1 gradient between marrow and blood might be useful as a hematopoietic stem cell mobilizing strategy [69, 70].

The disruption of the CXCR4/SDF-1α axis leads to the rapid mobilization of HSPCs from their original niche in BM [62, 71]. HPSCs migrate along a SDF-1α concentration gradient [27]. We predicted that SDF-1α of the injured liver promotes HPSC migration toward the liver via its receptors. In the current study, during the process of CCl4-induced liver injury, we detected a significant downregulation of SDF-1α gene expression in the BM and its significant upregulation in the liver with a positive concentration gradient towards the liver. These findings were associated with a significant concomitant elevation of the percent of CD34 + cells in the blood and the liver. SDF-1α decrease in BM and/or increase in peripheral blood can result in mobilization of stem cells towards the blood according to the concentration gradient between SDF-1α in both liver and BM and disruption of the SDF-1α axis at the bone marrow. HPSCs niche allows HPSCs to exit from the BM and migrate into the circulation according to the concentration gradient of SDF-1α [62]. The increased SDF-1α production in liver tissue after chronic liver injury and the decrease in its level of expression in bone marrow can in turn stimulate HPSC trafficking to the liver along the concentration gradient of SDF-1α [27]. The homing of these cells to the wounded liver is further facilitated by the SDF-1α concentration gradient, which is also implicated in the trafficking of cells out of the bone marrow [72].

In the current study, there was a clear correlation between the gene expression of SDF-1α in BM and CD34 + cells in the peripheral blood. While perhaps there are many of the proposed mechanistic pathways for CD34 + cell mobilization, the CXCR4/ SDF-1α axis appears to be the most important one for HPSCs mobilization [57].

The current finding of the enhanced CD34 + cell percentage in the peripheral blood was in line with Kong et al. [73] studies that reported an increase in the level of circulation CD34 + in the peripheral blood in response to hepatic injury. An increased level of peripheral blood HPSCs was also observed following extensive liver resection [74] and in patients with alcoholic hepatitis [16] with variability in the extent of their mobilization into the circulation according to the degree of the liver injury, which is consistent with our findings.

A fibroproliferative disease may emerge from the misdirection of the wound-healing process caused by excessive SDF-1α signaling with CXCR4 [75]. In a study of skin lesions, [76] suggested that the possibility of a reversible restoration of the activity of the residing fibroblasts is suggested by the downregulation of such an axis. To our knowledge, no previous study was performed to explore the change in SDF-1α, CD34 + cells, and CXCR4 receptor expression during the recovery process of liver fibrosis. In the resolution group, we observed a significant upregulation of SDF-1α gene expression in the BM and its significant downregulation in the liver, associated with a concomitant significant reduction of CD34 + cell percentage in peripheral blood and the liver. The restoration of the SDF-1α positive gradient towards the BM could limit the mobility of CD34 + cells toward the peripheral blood. Meanwhile, the decreased percent of CD34 + cells in the liver during the resolution phase could be due to their participation in hepatic repair after injury by either transdifferentiation of CD34 + to hepatocytes or fusion with the degenerated hepatocytes [77, 78]. Another study was done by [79] who found that the Granulocyte Colony Stimulating factor which stimulates the production of CD34 + HPSCs by the bone marrow, could contribute to the reduction of liver fibrosis induced by CCL4.

Many investigations in human patients and animal models suggested the involvement of SDF-1α –dependent and stellate cell activation pathways. SDF-1α binds to CXCR4 receptors on HSCs, thereby inducing HSC activation, proliferation, and production of collagen, which perpetuates fibrosis [80]. In agreement with this, we detected that the upregulated SDF-1α expression in the liver was accompanied by progressive and significant enhanced expression of CXCR4 receptors in the liver and the percentage of hepatic fibrosis area. SDF-1α promotes the activation of CXCR4 in the stellate cell population, increases the generation of reactive oxygen species in HSCs, and increases the expression of genes linked to fibrogenesis [4]. On the contrary, in the spontaneous recovery phase, we detected a downregulation of the elevated SDF-1α expression in the liver, accompanied by a progressive significant decrease in the expression of CXCR4 receptors in the liver and the percentage of the area of hepatic fibrosis, which may be due to the conversion and settling of CXCR4-positive cells as resident fibroblasts lose their phenotype over time [76].

Since the molecular basis of liver fibrosis is so complex, characterization of the condition is crucial for choosing treatment options and determining prognosis. [21]. While liver biopsy is the accepted method for determining liver fibrosis, it has significant drawbacks, such as being intrusive [25]. More reliable and non-invasive techniques for the detection and staging of liver fibrosis are becoming more and more necessary.

Interestingly, to our knowledge, our findings were the first to report a statistically substantial positive correlation of large strength between the percent of CD34 + cells in the peripheral blood and bone marrow, transaminases serum level and ratio, the expression of CXCR4 receptors in the liver, the level of SDF-1α in the liver and its concentration gradient with the liver fibrosis area percentage, and a statistically substantial negative correlation of large strength between SDF-1α in the BM and the liver fibrosis area percentage.

In addition, SDF-1α in the liver showed a negative correlation with its level in the bone marrow and a positive relationship with CXCR4 receptor expression in the liver. Finally, CXCR4 receptor expression in the liver showed a positive correlation with CD34 + cells in the blood and the concentration gradient for SDF-1α.

In conclusion, in the CCl4 rat model, there was a substantial correlation between the changes in fibrosis area percentage and the changes in CD34+, SDF-1α, and CXCR4 during the process of liver fibrosis treatment and also during the spontaneous recovery period. Scoring methods need to utilize one or more of these parameters to evaluate the efficiency of anti-fibrotic drugs in the treatment of hepatic fibrosis and the prognosis of liver illness. Additionally, it is hypothesized that the CXCR4/ SDF-1 axis that participates in liver fibrosis, can at the same time stimulate the migration & homing of HPSCs to the injured liver. Accordingly, further studies are needed to assess the role of these migrated CD34 + HPSCs and whether they are involved in the process of fibrosis or regeneration through the detection of the distribution and proportion of HPSCs and HSCs in fibrotic liver.

Further investigations are still needed to explore the details of the changes in these parameters with each degree of early and late liver fibrosis before approving them as potential biomarkers for monitoring liver fibrosis progression.

The main limitations of the research include the short time of the recovery period, as its prolongation could have provided additional information about the levels of the studied parameters during the regression. Furthermore, blocking the SDF-1α by knocking it down or using therapeutic agents would be useful to explore more informative monitoring of the process of liver fibrosis progression and regression.

## Data Availability

All data generated or analyzed during this study are included in this published article.
